# Associations of comprehensive dietary antioxidant index with postmenopausal femur bone mineral density and osteoporosis: data from the national health and nutrition examination survey

**DOI:** 10.3389/fnut.2025.1526532

**Published:** 2025-01-29

**Authors:** Jianbo Sun, Jie Wang, Wu Hu, He Huang, Hongmou Zhao

**Affiliations:** ^1^Department of Foot and Ankle Surgery, Honghui Hospital of Xi'an Jiaotong University, Xi'an, Shaanxi, China; ^2^Department of Orthopaedics, Tongyong Medical Xi'an Hospital, Xi'an, Shaanxi, China

**Keywords:** comprehensive dietary antioxidant index, antioxidant, bone mineral density, osteoporosis, American postmenopausal women

## Abstract

**Objectives:**

The study aimed to explore associations of the comprehensive dietary antioxidant index (CDAI) with femur bone mineral density (BMD) and risk of osteoporosis in American postmenopausal women.

**Methods:**

A total of 2,862 participants from the National Health and Nutrition Examination Survey were included in this study. The relationship between the CDAI and femur BMD was assessed via weighted multivariate linear regression model. The odds ratios (ORs) and 95% confidence intervals (95% CIs) for the association between the CDAI and the risk of osteoporosis was assessed by weighted logistic regression model. Moreover, the nonlinear relationship was also characterized by smooth curve fitting and weighted generalized additive model. The two-piecewise linear regression models and a recursive algorithm were used to find the inflection points.

**Results:**

After adjusting all covariates, the weighted multivariable linear regression models demonstrated that the CDAI was positively correlated with femur BMD. Moreover, there were nonlinear relationships between CDAI and risk of osteoporosis. In the age below 70 years, the risk of osteoporosis decreased to 60.6 and 92.2% with each unit increase in CDAI value before and after the inflection point (−2.268), respectively. In the 70 years or older, the risk of osteoporosis decreased to 80.4% with each unit increase in CDAI value before the inflection point (−1.479). The weighted logistic regression model demonstrated that compared to the first tertile of CDAI, the highest tertile of CDAI was significantly associated with a lower risk of osteoporosis, with ORs of 0.375 (95% CI 0.284, 0.495) for individuals under the age of 70, and 0.626 (95% CI 0.481, 0.814) for individuals aged 70 or above.

**Conclusion:**

The present study indicated that postmenopausal women with higher CDAI scores have a lower risk of osteoporosis. In addition, there is a non-linear relationship between CDAI and the risk of osteoporosis. This finding suggests that the adoption of a comprehensive antioxidant dietary structure represented by high CDAI scores may have a positive impact on the prevention and management of osteoporosis in postmenopausal women. Particularly for those with lower CDAI scores, early screening and intervention for osteoporosis may be necessary.

## 1 Introduction

Postmenopausal osteoporosis is the most common type of osteoporosis and affects approximately 50% of postmenopausal women worldwide (1). Patients are prone to fragility fractures due to microarchitectural deterioration of bone tissue, decreased bone mineral density (BMD), and increased bone fragility (2, 3). In the United States (U.S.), 1.5 million fractures are caused by osteoporosis each year, most of which occur in postmenopausal women (1). Especially, hip fractures that occur in postmenopausal women can be devastating and are a growing problem due to an aging population (1, 4). Because of the high incidence and serious complications of postmenopausal osteoporosis, it is of great significance to identify and control disease-related risk factors.

In recent years, oxidative stress response has been demonstrated to play an important role in the development and progression of osteoporosis (5, 6). Under normal physiological conditions, reactive oxygen species (ROS) products are produced in the mitochondrial respiratory chain of aerobic cells. Abnormal increases in ROS products due to various causes can lead to oxidative stress responses, which promote bone mass loss by increasing inflammatory responses, inhibiting osteoblast survival and differentiation, as well as activating osteoclast formation and bone resorption (7, 8). It has been recognized that intake of fruits and vegetables rich in antioxidants can suppress oxidative stress and reduce bone mass loss and risk of osteoporosis in postmenopausal women. Several epidemiological literatures have reported that BMD was positively associated with the intake of single food-based antioxidants, including vitamin A, C, E, manganese, selenium and zinc (9–13). However, the results are controversial. There were still studies have showed that serum concentrations of vitamin A, C and E are not associated with BMD in postmenopausal women, or even have an inverse relationship (14–18). This phenomenon may be explained in part by the presence of multiple antioxidants in the diet, which together exert cumulative and synergistic effects (19). Although a single antioxidant may play an active role in the etiology of osteoporosis, it does not represent the total intake of antioxidants in an individual. Therefore, when assessing the effect of antioxidant intake on BMD and osteoporosis risk in the postmenopausal population, it may be more appropriate to comprehensively incorporate multiple antioxidants and construct a concept of a comprehensive antioxidant network (20).

The composite dietary antioxidant index (CDAI) is a composite scoring tool for individuals based on a variety of common dietary antioxidant intakes, including vitamin A, C, E, manganese, selenium and zinc, and represents the total intake level of dietary antioxidants for an individual (21, 22). In addition, the development of CDAI also took into account the combined effect of anti-inflammatory, which has been shown to be negatively correlated with the levels of bone loss-related inflammatory factors such as IL-1β and TNF-*α* in individuals (23–25). However, previous research reports on CDAI were mainly in the field of oncology, such as its inverse association with lung and colorectal cancer risk (21, 22). The studies exploring the link between CDAI and femoral BMD, as well as the risk of osteoporosis is currently insufficient. In particular, it has not been deeply explored in the special population of postmenopausal women. Compared to the general adult population, aging and estrogen deficiency make ROS accumulation more significant in this population (3, 26). And given the high incidence and devastating outcomes of osteoporosis in this population (1), it may be essential to explore the relationship between a comprehensive antioxidant diet and bone health in this population. Therefore, the purpose of the current study was to further explore this relationship in the specific demographic of U.S. postmenopausal women.

## 2 Materials and methods

### 2.1 Study population

**Figure 1:**
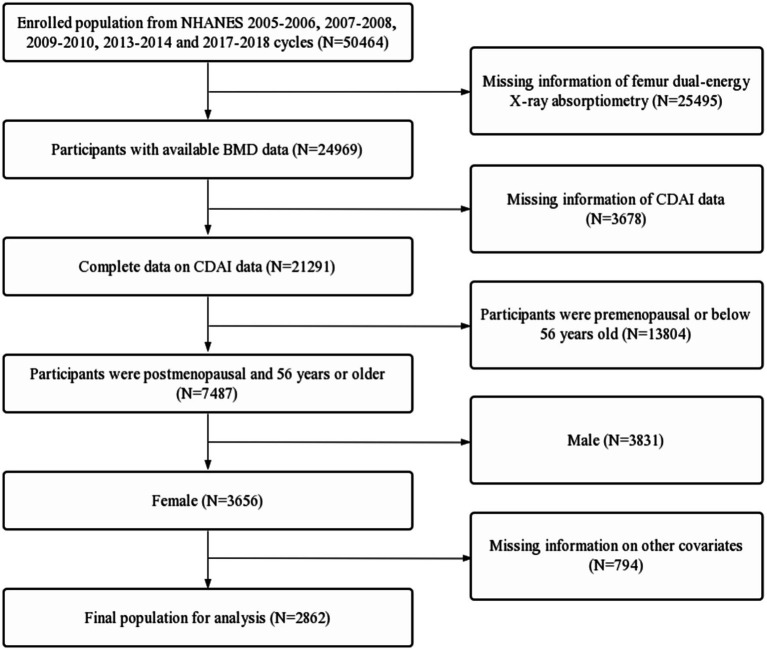
Flow diagram of inclusion criteria and exclusion criteria. NHANES, National Health and Nutrition Examination Survey; CDAI, composite dietary antioxidant index; BMD, bone mineral density.

The data used in this study came from the continuous National Health and Nutrition Examination Survey (NHANES) 2005–2006, 2007–2008, 2009–2010, 2013–2014 and 2017–2018 cycles. NHANES data were collected from a nationally representative sample of the U.S. civilian population via a multilevel probability design. NHANES was approved by the ethical review board of the National Center for Health Statistics, and all participants provided written informed consent. The inclusion criteria were as follows: (1) population from the continuous 2005–2006, 2007–2008, 2009–2010, 2013–2014 and 2017–2018 cycles in the NHANES database; (2) participants with available BMD data; (3) complete data on CDAI; (3) participants were postmenopausal and 56 years or older; (4) female. The exclusion criteria were as follows: (1) missing information of femur dual-energy X-ray absorptiometry; (2) missing information of CDAI data; (3) participants were premenopausal or below 56 years old; (4) male; (5) missing information on covariates. The flow chart of inclusion and exclusion process for this study were detailed in Figure 1.

### 2.2 BMD and definition of osteoporosis

BMD of the total femur, femoral neck, trochanter, and intertrochanter were assessed using dual-energy X-ray absorptiometry scans from a Hologic QDR-4500A fan-beam densitometer (Hologic, Inc., Bedford, Massachusetts). According to the World Health Organization classification criteria, BMD values in any femoral region lower than −2.5 standard deviations of the young adult reference group can be defined as osteoporosis. Specific thresholds were 0.68 g/cm^2^, 0.59 g/cm^2^, 0.49 g/cm^2^, and 0.78 g/cm^2^ for the total femur, femoral neck, trochanter, and intertrochanter, respectively (27).

### 2.3 Dietary assessment and CDAI calculation

Dietary data were extracted from two NHANES 24-h recall interviews. The first interview was carried out at the Mobile Examination Center (MEC), and the second was carried out by telephone 3 to 10 days later. The mean value of the two 24-h recall data was determined as needed dietary data in this study. Based on the dietary intake of six antioxidants including zinc, selenium, carotenoids and vitamins A, C and E, the CDAI was calculated as follows (22, 25):


$$\mathrm{CDAI}=\overset{n=6}{\underset{i=1}{\Sigma }}\frac{x_{i}-\mu_{i}}{s_{i}}$$

In the above formula, i represented antioxidant; x_i_ represented the daily intake of i; μ_i_ represented the mean value of x_i_ in the whole cohort; s_i_ represented the standard deviation (SD) for μ_i_.

### 2.4 Covariates

Based on the previous literatures and clinical experience, the selected covariates were obtained as follows:

Demographic data: age (<70 years, ≥70 years), race (Mexican Americans, other Hispanic, non-Hispanic White, non-Hispanic Black, other race), educational level (less than 9th grade, 9–11th grade, high school, some college, college graduate), marital status (married, widowed, divorced, separated, never married, living with partner), and poverty income ratio (PIR; <1, 1–3, ≥3);

Examination data: body mass index (BMI; <25, 25–30, ≥30);

Dietary data: energy intakes (the mean value of the two 24-h recall data).

Questionnaire data: arthritis (yes or no), alcohol consumption (drink/d), smoked at least 100 cigarettes (yes or no), ever use prednisone or cortisone daily (yes or no), ever use female hormones (yes or no), had a hysterectomy (yes or no), moderate or vigorous activity (yes or no), and postmenopausal period, which was calculated by the age when taking the questionnaire minus age at last menstrual period.

Comprehensive data: hypertension status (yes or no) and diabetes status (yes, no or borderline). hypertension status was defined according to the following criterions: doctor told you have hypertension, use of hypertension drugs, or mean value of 3 measured diastolic blood pressure ≥ 90 mmHg or the mean value of 3 measured systolic pressure ≥ 140 mmHg (The reading with zero is not used to calculate the diastolic average, and if only one blood pressure reading was obtained, that reading is the average). Besides, estimated glomerular filtration rate (eGFR) was calculated based on the Chronic Kidney Disease Epidemiology Collaboration (CKD-EPI) equation (28).

### 2.5 Statistical analysis

Sampling weights were used in all analyses based on the weight selection criteria of NHANES. Data of continuous and categorical variables were, respectively, described by the mean and proportion. The differences of categorical variables between the osteoporosis and non-osteoporosis groups were compared by Chi-square test. Moreover, a Shapiro–Wilk test was performed to test the normality for the continuous variables. For continuous variables that conformed the normal distribution, a Student’s t-test was used to compare the differences between the osteoporosis and non-osteoporosis groups. For continuous variables that did not conform to the normal distribution, a Wilcoxon rank sum test was used. The relationships between the CDAI and femur BMD were assessed via weighted multivariate linear regression model.

The odds ratios (ORs) and 95% confidence intervals (95% CIs) for the associations between the CDAI and the risk of osteoporosis were assessed by weighted logistic regression model. In addition, the nonlinear relationship was characterized by smooth curve fitting (SCF) and weighted generalized additive model (GAM). The two-piecewise linear regression models and a recursive algorithm were used to find the inflection points, at which the data trend of the nonlinear relationship curve changes significantly. To ensure the robustness of data analysis, the value of CDAI was categorized based on quartiles, and tests for linear trends were performed. All steps described above were also performed to evaluate the relationships between the categorized CDAI and femur BMD and the risk of osteoporosis. Model 1 was adjusted for no covariates. Model 2 was adjusted for age (if applicable) and race. Model 3 was adjusted covariates including age, race, BMI, PIR, educational level, marital status, diabetes status, hypertension status, arthritis, eGFR, ever use prednisone or cortisone daily, smoked at least 100 cigarettes, alcohol consumption, ever use female hormones, had a hysterectomy, moderate or vigorous activity, and postmenopausal period.

All analyses were performed via R software (4.0.3) and EmpowerStats (2.0). A two-sided *p* value <0.05 was considered to have statistical significance.

## 3 Results

### 3.1 Baseline characteristics of participants

A total of 2,862 participants was included in this study, which far more than the number of independent variables and covariables. According to the relevant statistical principles (29, 30), it was sufficient to support the statistical analysis in this study. Based on the diagnosis criteria of osteoporosis (27), the prevalence of osteoporosis was 20.6% (589/2862) in the present study. In the osteoporosis group, participants had significantly lower values of CDAI (0.9 ± 2.9 vs. 0.5 ± 3.1, *p* = 0.003) but longer postmenopausal years (18.7 ± 10.9 vs. 24.7 ± 11.5, *p* < 0.001). Moreover, participants with osteoporosis were more likely to be older, thinner, poorer, lower educational level, widowed. The percentage of participants who had a hysterectomy, smoked more cigarettes, had less activity, and worse kidney function were significantly higher in the osteoporosis group (*p* < 0.050; Table 1).

**Table 1:** Weighted characteristics of the study population.

	Non-osteoporosis (*N* = 2,273, 79.4%)	Osteoporosis (*N* = 589, 20.6%)	*p* value
CDAI (mean ± SD)	0.9 ± 2.9	0.5 ± 3.1	0.003
Age (*N*, %)			<0.001
<70	1,589, 69.9	274, 46.5	
≥ 70	684, 30.1	315, 53.5	
Race (*N*, %)			<0.001
Mexican Americans	86, 3.8	16, 2.8	
Other Hispanic	65, 2.9	21, 3.6	
Non-Hispanic White	1834, 80.7	497, 84.4	
Non-Hispanic Black	193, 8.5	21, 3.5	
Other race	95, 4.2	34, 5.7	
BMI (*N*, %)			<0.001
<25	566, 24.9	301, 51.1	
≥ 25, <30	771, 33.9	186, 31.6	
≥ 30	936, 41.2	102, 17.4	
PIR (*N*, %)			<0.001
<1	173, 7.6	52, 8.9	
≥ 1, <3	843, 37.1	286, 48.5	
≥ 3	1,257, 55.3	251, 42.6	
Educational level (*N*, %)			<0.001
Less than 9th grade	91, 4.0	36, 6.1	
9–11th grade	209, 9.2	77, 13.0	
High school	623, 27.4	179, 30.4	
Some college	680, 29.9	171, 29.0	
College graduate	670, 29.5	126, 21.4	
Marital status (*N*, %)			<0.001
Married	1,357, 59.7	269, 45.7	
Widowed	402, 17.7	191, 32.5	
Divorced	366, 16.1	101, 17.0	
Separated	27, 1.2	6, 1.0	
Never married	83, 3.7	16, 2.7	
Living with partner	38, 1.7	6, 1.1	
Diabetes status (*N*, %)			0.012
Yes	492, 21.6	98, 16.7	
No	1,561, 68.7	419, 71.2	
Borderline	220, 9.7	72, 12.2	
Hypertension status (*N*, %)			0.918
Yes	1,266, 55.7	330, 56.0	
No	1,007, 44.3	259, 44.0	
Arthritis (*N*, %)			0.647
Yes	1,255, 55.2	319, 54.1	
No	1,018, 44.8	270, 45.9	
eGFR (mL/(min 1.73 m^2^), *N*, %)			<0.001
<60	396, 17.4	150, 25.4	
≥ 60, <90	1,268, 55.8	316, 53.7	
≥ 90	609, 26.8	123, 20.9	
Ever use prednisone or cortisone daily (*N*, %)			0.798
Yes	189, 8.3	51, 8.6	
No	2084, 91.7	538, 91.4	
Smoked at least 100 cigarettes (*N*, %)			0.001
Yes	902, 39.7	277, 47.0	
No	1,371, 60.3	312, 53.0	
Alcohol consumption (drink/d, mean ± SD)	0.8 ± 2.0	0.6 ± 1.5	0.589
Had a hysterectomy (*N*, %)			0.003
Yes	932, 41.0	281, 47.7	
No	1,341, 59.0	308, 52.3	
Ever use female hormones (*N*, %)			<0.001
Yes	1,150, 50.6	252, 42.7	
No	1,123, 49.4	337, 57.3	
Postmenopausal period (years, mean ± SD)	18.7 ± 10.9	24.7 ± 11.5	<0.001
Moderate or vigorous activity (*N*, %)			0.002
Yes	1,184, 52.1	265, 45.0	
No	1,089, 47.9	324, 55.0	
Energy intake (kcal, mean ± SD)	1693.0 ± 543.3	1651.6 ± 583.9	0.1046
Total femur BMD (g/cm^2^, mean ± SD)	0.9 ± 0.1	0.7 ± 0.1	<0.001
Femur neck BMD (g/cm^2^, mean ± SD)	0.7 ± 0.1	0.6 ± 0.1	<0.001
Trochanter BMD (g/cm^2^, mean ± SD)	0.7 ± 0.1	0.5 ± 0.1	<0.001
Intertrochanter BMD (g/cm^2^, mean ± SD)	1.1 ± 0.1	0.8 ± 0.1	<0.001

CDAI, composite dietary antioxidant index; BMD, bone mineral density; PIR, poverty income ratio; BMI, body mass index; eGFR, estimated glomerular filtration rate; SD, standard deviation; %, weighted percentage.

### 3.2 Associations of CDAI with femur BMD

**Figure 2:**
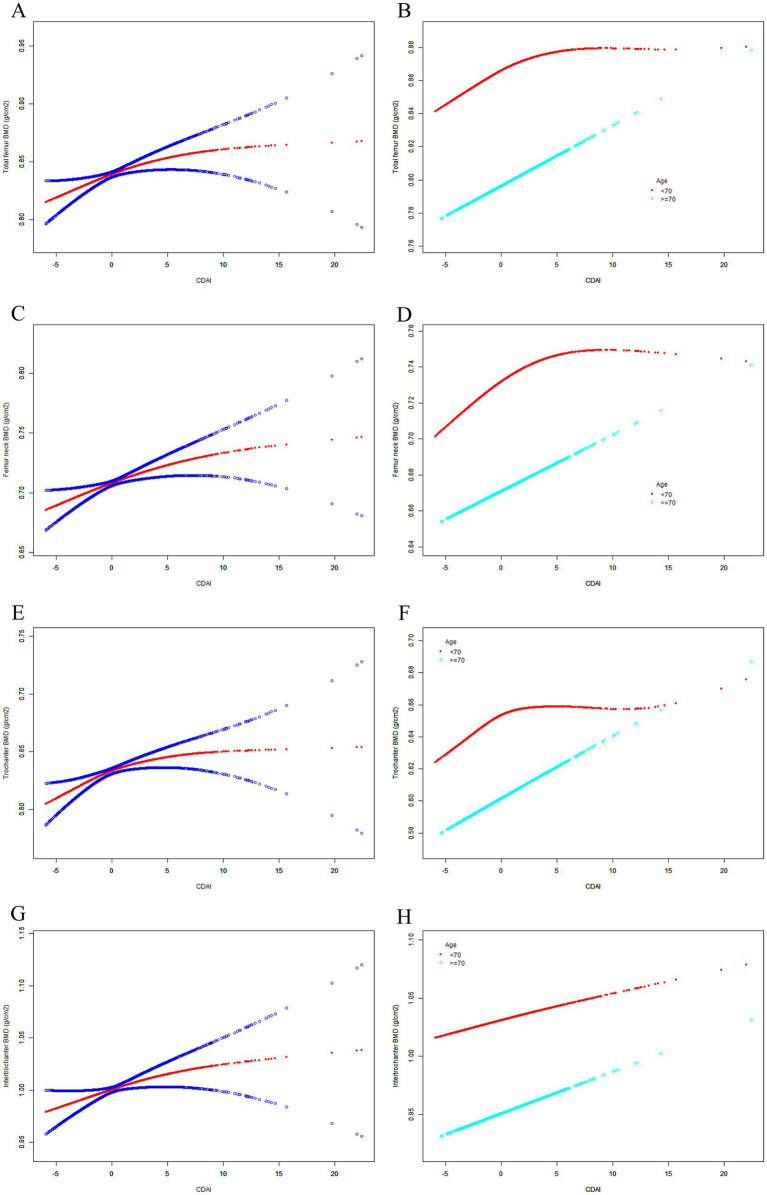
The SCF for the association between CDAI and femur BMD. Age (in A, C, E, G), race, BMI, educational level, marital status, PIR, smoked at least 100 cigarettes, alcohol consumption, energy intake, hypertension status, diabetes status, arthritis, eGFR, ever use prednisone or cortisone daily, ever use female hormones, had a hysterectomy, moderate or vigorous activity, and postmenopausal period were adjusted. **(A)** Total femur BMD; **(B)** Femur neck BMD; **(C)** Trochanter BMD; **(D)** Intertrochanter BMD; SCF, smooth curve fit; BMD, bone mineral density; CDAI, composite dietary antioxidant index; PIR, poverty income ratio; BMI, body mass index.

The weighted multivariate linear regression model showed that the CDAI generally showed a positive correlation trend with femur BMD in Model 1. After adjusting for confounding factors in Model 2 (age and race) and Model 3 (all covariates), the trends remained stable (Table 2). In addition, the SCF and GAM showed there was a non-linear relationship between CDAI and both femur BMD (Table 3; Figure 2). When stratified by age, the CDAI also showed a positive correlation trend with femur BMD in different age groups. The trends were not significantly affected by adjustment for covariates (Table 2). After adjusting for all covariates in the age below 70 years, there were nonlinear relationships between CDAI and other part of femur BMD except for intertrochanter BMD (Table 3; Figure 2). After adjusting for all covariates in the 70 years or older, only the nonlinear relationship between CDAI and trochanter BMD was found (Table 3; Figure 2).

**Table 2:** Associations of the CDAI with femur BMD and the risk of osteoporosis.

	Age < 70 β (95% CI) *p* value	Age ≥ 70 β (95% CI) *p* value	Total
Total femur BMD (g/cm^2^)			
Model 1	0.001 (−0.001, 0.003) 0.284	0.003 (0.001, 0.006) 0.017	0.002 (0.000, 0.004) 0.026
Model 2	0.002 (−0.000, 0.004) 0.086	0.004 (0.001, 0.007) 0.008	0.002 (0.001, 0.004) 0.003
Model 3	0.001 (−0.001, 0.004) 0.347	0.004 (0.001, 0.008) 0.012	0.002 (0.000, 0.004) 0.025
Femur neck BMD (g/cm^2^)			
Model 1	0.000 (−0.002, 0.002) 0.819	0.002 (−0.001, 0.004) 0.222	0.001 (−0.001, 0.002) 0.393
Model 2	0.001 (−0.001, 0.003) 0.248	0.002 (−0.000, 0.005) 0.079	0.001 (−0.000, 0.003) 0.053
Model 3	0.002 (−0.000, 0.004) 0.116	0.004 (0.001, 0.007) 0.019	0.003 (0.001, 0.005) 0.006
Trochanter BMD (g/cm^2^)			
Model 1	0.001 (−0.001, 0.002) 0.464	0.004 (0.002, 0.006) <0.001	0.002 (0.000, 0.003) 0.011
Model 2	0.001 (−0.001, 0.003) 0.215	0.004 (0.002, 0.006) <0.001	0.002 (0.001, 0.003) 0.003
Model 3	0.000 (−0.002, 0.002) 0.855	0.004 (0.002, 0.007) 0.003	0.002 (−0.000, 0.003) 0.055
Intertrochanter BMD (g/cm^2^)			
Model 1	0.002 (−0.001, 0.004) 0.198	0.003 (−0.000, 0.007) 0.058	0.002 (0.000, 0.004) 0.032
Model 2	0.002 (−0.000, 0.005) 0.063	0.004 (0.000, 0.007) 0.033	0.003 (0.001, 0.005) 0.006
Model 3	0.002 (−0.001, 0.005) 0.264	0.004 (−0.000, 0.008) 0.070	0.003 (0.000, 0.005) 0.049
Osteoporosis			
Model 1	0.938 (0.916, 0.960) <0.001	0.972 (0.949, 0.995) 0.017	0.954 (0.938, 0.970) <0.001
Model 2	0.930 (0.908, 0.952) <0.001	0.964 (0.941, 0.988) 0.003	0.946 (0.930, 0.962) <0.001
Model 3	0.900 (0.868, 0.933) <0.001	0.955 (0.922, 0.989) 0.009	0.924 (0.901, 0.947) <0.001

Model 1: no covariates were adjusted. Model 2: age (if applicable) and race were adjusted. Model 3: age (if applicable), race, BMI, educational level, marital status, PIR, smoked at least 100 cigarettes, alcohol consumption, energy intake, hypertension status, diabetes status, arthritis, eGFR, ever use prednisone or cortisone daily, ever use female hormones, had a hysterectomy, moderate or vigorous activity, and postmenopausal period were adjusted. CDAI, composite dietary antioxidant index; BMD, bone mineral density; PIR, poverty income ratio; BMI, body mass index; eGFR, estimated glomerular filtration rate.

**Table 3:** Two-piecewise linear regression models of CDAI on femur BMD and the risk of osteoporosis.

	Age < 70 β (95% CI) *p* value	Age ≥ 70 β (95% CI) *p* value	Total
Total femur BMD (g/cm^2^)			
Inflection point	−1.964	−0.626	−0.626
< Inflection point	0.013 (0.002, 0.025) 0.027	0.012 (0.002, 0.022) 0.018	0.009 (0.003, 0.015) 0.001
> Inflection point	0.000 (−0.002, 0.003) 0.787	0.003 (−0.001, 0.007) 0.153	0.001 (−0.001, 0.003) 0.438
P_L_	0.038	0.105	0.010
Femur neck BMD (g/cm^2^)			
Inflection point	−1.887	−1.515	−1.887
< Inflection point	0.016 (0.005, 0.027) 0.005	0.010 (−0.003, 0.023) 0.117	0.015 (0.006, 0.024) 0.001
> Inflection point	0.001 (−0.002, 0.004) 0.489	0.003 (−0.000, 0.006) 0.070	0.002 (−0.000, 0.004) 0.091
P_L_	0.010	0.279	0.004
Trochanter BMD (g/cm^2^)			
Inflection point	−0.632	−1.470	−1.529
< Inflection point	0.008 (0.002, 0.013) 0.011	0.016 (0.004, 0.028) 0.007	0.013 (0.006, 0.019) <0.001
> Inflection point	−0.001 (−0.004, 0.001) 0.278	0.003 (0.000, 0.006) 0.043	0.001 (−0.001, 0.002) 0.554
P_L_	0.007	0.040	<0.001
Intertrochanter BMD (g/cm^2^)			
Inflection point	3.693	0.437	3.465
< Inflection point	0.004 (−0.000, 0.008) 0.053	0.010 (0.001, 0.019) 0.030	0.005 (0.002, 0.008) 0.004
> Inflection point	−0.002 (−0.008, 0.003) 0.406	0.001 (−0.004, 0.007) 0.621	−0.002 (−0.007, 0.003) 0.453
P_L_	0.087	0.127	0.032
Osteoporosis			
Inflection point	−2.268	−1.479	−2.031
< Inflection point	0.606 (0.513, 0.715) <0.001	0.804 (0.704, 0.918) 0.001	0.695 (0.623, 0.775) <0.001
> Inflection point	0.922 (0.889, 0.957) <0.001	0.973 (0.938, 1.010) 0.155	0.944 (0.920, 0.968) <0.001
P_L_	<0.001	0.009	<0.001

Age (if applicable), race, BMI, educational level, marital status, PIR, smoked at least 100 cigarettes, alcohol consumption, energy intake, hypertension status, diabetes status, arthritis, eGFR, ever use prednisone or cortisone daily, ever use female hormones, had a hysterectomy, moderate or vigorous activity, and postmenopausal period were adjusted. CDAI, composite dietary antioxidant index; BMD, bone mineral density; PIR, poverty income ratio; BMI, body mass index; eGFR, estimated glomerular filtration rate; P_L_, Log likelihood ratio.

### 3.3 Associations of CDAI with osteoporosis

**Figure 3:**
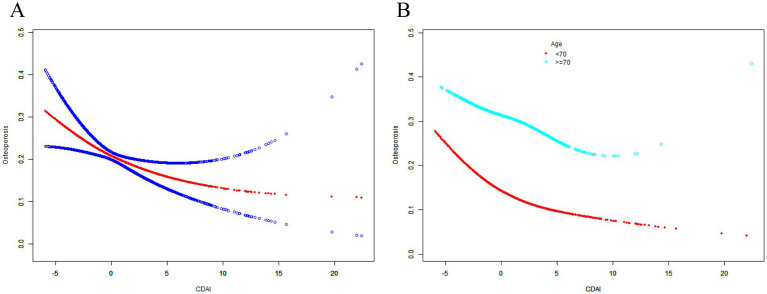
The associations between CDAI and the risk of osteoporosis. Age (in A), race, BMI, educational level, marital status, PIR, smoked at least 100 cigarettes, alcohol consumption, energy intake, hypertension status, diabetes status, arthritis, eGFR, ever use prednisone or cortisone daily, ever use female hormones, had a hysterectomy, moderate or vigorous activity, and postmenopausal period were adjusted. SCF, smooth curve fit; BMD, bone mineral density; CDAI, composite dietary antioxidant index; PIR, poverty income ratio; BMI, body mass index.

Based on the results of weighted logistic regression model analysis, this study found the CDAI generally showed a robust negative correlation with the risk of osteoporosis, and the trend was not significantly affected by covariate adjustment (Table 2). After adjusting for all covariates, the risk of osteoporosis decreased to 92.4%, which appeared to have a weak statistical power (Table 2). Moreover, the non-linear relationship between CDAI and the risk of osteoporosis was also be characterized by the SCF and GAM (Table 3; Figure 3). After further in-depth analysis of the two-piecewise linear regression, the statistical power of the two associations became clearer. Before the inflection point, the correlation between the two was significantly stronger than after the inflection point. After adjusting for all covariates, the risk of osteoporosis decreased to 69.5 and 94.4% with each unit increase in CDAI value before and after the inflection point (−2.031; Table 3), respectively. When CDAI was classified according to quartiles, the association between CDAI and osteoporosis was more significant. The ORs between the risk of osteoporosis and CDAI values across quintiles 2 (CDAI.Q2), 3 (CDAI.Q3) and 4 (CDAI.Q4) compared with quintile 1 (CDAI.Q1) were, respectively, 0.750 (95% CI 0.646, 0.872; *p* < 0.001), 0.706 (95% CI 0.602, 0.829; *p* < 0.001) and 0.487 (95% CI 0.405, 0.586; *p* < 0.001) in Model 3 (Table 4). The trend test also showed that with the increase of CDAI quartiles groups, the risk of osteoporosis decreased (P for trend <0.001; Table 4).

**Table 4:** Associations of the CDAI.Q4 with the risk of osteoporosis.

	Age < 70 β (95% CI) *p* value	Age ≥ 70 β (95% CI) *p* value	Total
Osteoporosis			
Model 1			
Q1 (−5.903–−1.550)	Reference	Reference	Reference
Q2 (−1.546–0.069)	0.819 (0.684, 0.980) 0.029	0.771 (0.635, 0.937) 0.009	0.795 (0.696, 0.908) <0.001
Q3 (0.075–2.152)	0.664 (0.555, 0.795) <0.001	0.758 (0.624, 0.920) 0.005	0.706 (0.619, 0.806) <0.001
Q4 (2.158–22.417)	0.499 (0.414, 0.601) <0.001	0.747 (0.619, 0.901) 0.002	0.610 (0.535, 0.696) <0.001
Model 2			
Q1 (−5.903–−1.550)	Reference	Reference	Reference
Q2 (−1.546–0.069)	0.790 (0.659, 0.947) 0.011	0.745 (0.613, 0.906) 0.003	0.768 (0.672, 0.878) <0.001
Q3 (0.075–2.152)	0.627 (0.523, 0.752) <0.001	0.720 (0.592, 0.876) 0.001	0.669 (0.585, 0.764) <0.001
Q4 (2.158–22.417)	0.468 (0.388, 0.565) <0.001	0.701 (0.579, 0.848) <0.001	0.573 (0.502, 0.655) <0.001
Model 3			
Q1 (−5.903–−1.550)	Reference	Reference	Reference
Q2 (−1.546–0.069)	0.770 (0.622, 0.953) 0.016	0.691 (0.554, 0.861) 0.001	0.750 (0.646, 0.872) <0.001
Q3 (0.075–2.152)	0.716 (0.570, 0.900) 0.004	0.724 (0.572, 0.915) 0.007	0.706 (0.602, 0.829) <0.001
Q4 (2.158–22.417)	0.375 (0.284, 0.495) <0.001	0.626 (0.481, 0.814) <0.001	0.487 (0.405, 0.586) <0.001
P trend	<0.001	<0.001	<0.001

Model 1: no covariates were adjusted. Model 2: age (if applicable) and race were adjusted. Model 3: age (if applicable), race, BMI, educational level, marital status, PIR, smoked at least 100 cigarettes, alcohol consumption, energy intake, hypertension status, diabetes status, arthritis, eGFR, ever use prednisone or cortisone daily, ever use female hormones, had a hysterectomy, moderate or vigorous activity, and postmenopausal period were adjusted. CDAI, composite dietary antioxidant index; BMD, bone mineral density; PIR, poverty income ratio; BMI, body mass index; eGFR, estimated glomerular filtration rate.

When stratified by age, the CDAI also showed a negative correlation trend with the risk of osteoporosis in different age groups. After adjusting for all covariates in the age below 70 years, the risk of osteoporosis decreased to 60.6 and 92.2% with each unit increase in CDAI value before and after the inflection point (−2.268; Table 3; Figure 3B), respectively. Compared with CDAI.Q1, the OR between CDAI.Q4 and the risk of osteoporosis was 0.375 (95% CI 0.284, 0.495; *p* < 0.001) in Model 3 (Table 4). After adjusting for all covariates in the 70 years or older, the risk of osteoporosis decreased to 80.4% with each unit increase in CDAI value before the inflection point (−1.479; Table 3), but the association between the two after the inflection point was not statistically significant (*p* = 0.155, Table 3). Compared with CDAI.Q1, the OR between CDAI.Q4 and the risk of osteoporosis was 0.626 (95% CI 0.481, 0.814; p < 0.001) in Model 3 (Table 4). These results further highlight that an increase in CDAI may be associated with modest improvements in femur BMD, which may help reduce the risk of osteoporosis. It is interesting that the effect was even more pronounced in postmenopausal women younger than 70.

## 4 Discussion

Based on a representative sample of postmenopausal women in the U.S. from the NHANES database, we observed a positive correlation between the CDAI score and femoral bone density, and a negative correlation with the risk of osteoporosis. Subgroup analysis by age further solidified the consistency of this relationship. Our findings underscore the significance of a diet abundant in antioxidants for maintaining bone health within this demographic. Significantly, we identified a nonlinear relationship between the CDAI score and femoral BMD, as well as with the risk of osteoporosis, distinctly within the overall population and different age subgroups. Interestingly, the nonlinear relationship was more pronounced in the younger population (under 70 years old). Previous studies have primarily focused on the correlation analysis between CDAI and osteoporosis, without a thorough investigation into the specific linear or nonlinear relationships between the two (31–33). To the best of our knowledge, this is the first study in the specific population of American postmenopausal women that delved into the nonlinear relationship between CDAI and femoral BMD, and the risk of osteoporosis.

Relevant studies indicate that factors such as mitochondrial dysfunction and DNA damage caused by aging can result in excessive generation of ROS (34, 35). Additionally, estrogen possesses the antioxidant property of interrupting the chain reaction of free radicals (36). The decrease of estrogen in the body weakens this reaction and increases the accumulation of ROS (37). The elevated ROS level caused by the imbalance between ROS generation and elimination is an independent risk factor for osteoporosis (26). When the antioxidant balance within the body is disrupted, the intake of exogenous antioxidants can prevent or delay the development of osteoporosis (20, 38). It is widely known that aging and estrogen deficiency are significant physiological characteristics of postmenopausal women (3). Therefore, the intake of dietary antioxidants is of great significance for this population in preventing and delaying the progression of osteoporosis. In recent years, the impact of individual antioxidants on bone health has been thoroughly investigated. For instance, Chen et al. reported that higher dietary vitamin A and *β*-carotene levels were positively associated with BMD of Chinese adults (9). Malmir et al. suggested that increasing dietary vitamin C intake was associated with a lower risk of femoral neck and lumbar hip fractures and osteoporosis (39). Ruiz-Ramos and Michaelsson K et al. found that supplementation with ascorbic acid and *α*-tocopherol may help prevent or help treat age-related osteoporosis (12, 40). In addition, the study of Chen et al. proved that copper and zinc intake was associated with a lower incidence of osteoporosis in elderly hypertensive patients, which may be beneficial to bone health in elderly hypertensive patients (41). Grili et al. found that dietary selenium intake was negatively correlated with the risk of osteoporosis in postmenopausal women (42). However, as reported by Yang et al., vitamin E congeners *α*- and *γ*-tocopherol were not associated with bone turnover markers or BMD in perimenopausal and postmenopausal women, and did not have a theoretical anti-osteoporosis effect (15). Mangano et al. also reported that dietary vitamin C was not associated with BMD in Puerto Rican adults in Boston (11). Furthermore, Lionikaite et al. suggested that increased vitamin A intake was associated with decreased cortical bone mass and increased fracture risk in humans (16). Zhang et al. found that serum α-tocopherol concentration was inversely associated with bone mineral density in the elderly population in the United States, so it is not considered necessary to use vitamin E supplements as α-tocopherol to promote bone health (14). The controversial findings are largely attributed to the diversity and interactions of nutrients within foods. When investigating a single nutrient, it is challenging to account for the biases caused by the interplay of various nutrients in the diet. In recent years, nutritional research has shifted from focusing on the benefits of individual nutrients to emphasizing the advantages of the overall dietary structure (43, 44). With this in mind, we have further explored the relationship between bone health and a comprehensive score of CDAI, which incorporates multiple dietary antioxidants rather than a single antioxidant.

In this investigation, we performed a subgroup analysis among postmenopausal women, using the age of 70 as a demographic cutoff, to delve more deeply into the relationship between the CDAI and bone health within the pertinent population. Notably, the inverse association between CDAI and the risk of femoral osteoporosis was more pronounced in the younger cohort, those under the age of 70. Even after accounting for all confounding variables, this correlation remained robust. Previous studies of age-related oxidative stress may explain this phenomenon. Aging can lead to more serious mitochondrial dysfunction, DNA damage, and a more widespread pro-inflammatory microenvironment, which collectively may cause a more severe accumulation of ROS in the elderly compared to younger individuals. Consequently, even with the same intake of antioxidants, the elderly may have a weaker capacity to eliminate oxidative stress within their bodies compared to younger age groups (26, 44). In general, postmenopausal people may benefit from an antioxidant diet represented by a high CDAI score. On this basis, our study further revealed that the relationship between CDAI and the risk of osteoporosis was not only negatively correlated overall, but also had a deeper nonlinear correlation, which is of great significance for supplementing the previous relevant studies.

There are several advantages to this study. Firstly, this is the first study to characterize the non-linear relationship between CDAI and the risk of osteoporosis in postmenopausal women. Secondly, the data used in this study came from a large nationally representative database with a standardized data collection protocol, thereby reducing potential biases. Lastly, the study conducted subgroup analyses by age groups and trend tests after quartile classification of CDAI, enhancing the robustness of the data analysis. However, there are also limitations to this study. Firstly, its cross-sectional nature means that there may not be enough evidence to infer a causal relationship between the antioxidant diet structure represented by CDAI and osteoporosis. Secondly, the diet data collection in NHANES relies on questionnaires and interview surveys, which may be prone to recall bias. In addition, the antioxidants in this study were only limited to six including vitamins A, C, E, zinc, selenium and carotenoids, and the inclusion of other antioxidants such as manganese in the analysis was lacking (25). Lastly, although previous studies have reported the association between CDAI and bone loss-related inflammatory factors (23–25), this study was not able to verify this due to data limitations. Future studies, including prospective or laboratory research, are needed to explore and complement this further.

## 5 Conclusion

In summary, the results of this study indicated that postmenopausal women with higher CDAI scores have greater overall femoral BMD and a lower risk of osteoporosis. In addition, there is a non-linear relationship between CDAI and the risk of osteoporosis. Interestingly, for the people older than 70 years, the association between CDAI and the risk of femur osteoporosis was statistically significant only before the inflection point. This finding suggests that the adoption of a comprehensive antioxidant dietary structure represented by high CDAI scores may have a positive impact on the prevention and management of osteoporosis in postmenopausal women. Particularly for those with lower CDAI scores, early screening and intervention for osteoporosis may be necessary.

## Data Availability

The raw data supporting the conclusions of this article will be made available by the authors, without undue reservation.
